# Supplementary material for: Mature fault mechanics revealed by the highly efficient 2025 Mandalay earthquake
Source: Nat Commun. 2025 Dec 8;16:10937. doi: 10.1038/s41467-025-65942-2 (PMC12685957; doi:10.1038/s41467-025-65942-2)
Supplement: Supplementary file 1 — Supplementary Information [file 41467_2025_65942_MOESM1_ESM.pdf]

Supplementary Information for:

Mature fault mechanics revealed by the highly efficient 2025 Mandalay earthquake

Eric O. Lindsey[1], Yu-Ting Kuo[2], Yu Wang[3], Myo Thant[4,5,6], Tha Zin Htet Tin[5,6]

[1]Department of Earth and Planetary Sciences, The University of New Mexico, United States

[2]Department of Earth and Environment Sciences, National Chung Cheng University, Taiwan

[3]Department of Geosciences, National Taiwan University, Taiwan

[4]Myanmar Institute of Earth and Planetary Sciences, Myanmar

[5]Myanmar Earthquake Committee, Myanmar

[6]Department of Geology, University of Yangon, Myanmar

This file contains the following supplementary information:

- Supplementary Figures S1 – S23
- Supplementary Table S1
- Supplementary References

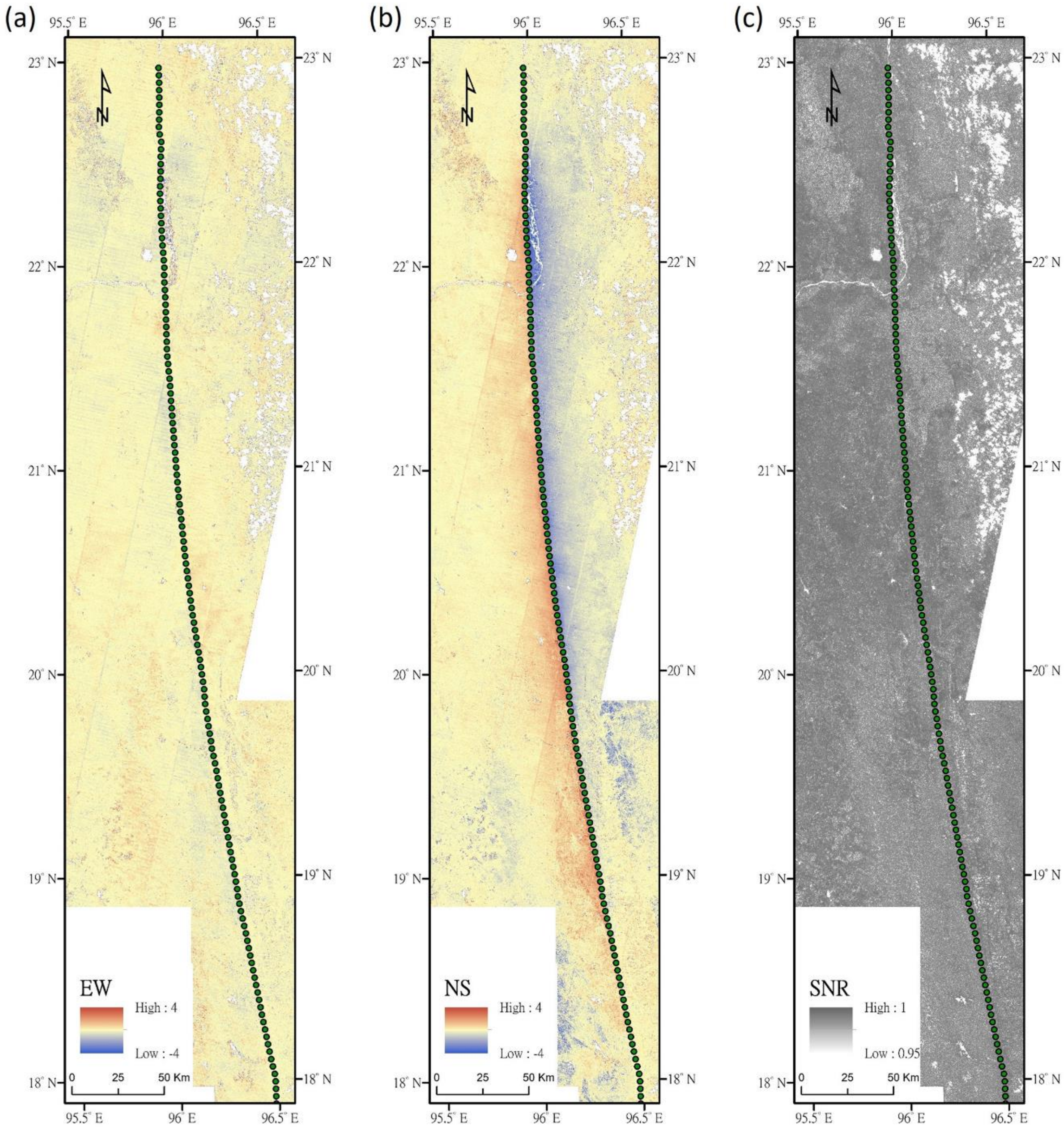

Supplementary Figure S1. Coseismic horizontal displacements calculated from Sentinel-2C imagery acquired on March 30 and April 1, 2025, with pre-earthquake reference images from March 2 and 20, 2025 by COSI-Corr technique (see Methods). (a) East-West component; (b) North-South component; (c) Signal-to-Noise ratio. Contains modified Copernicus Sentinel data [2025].

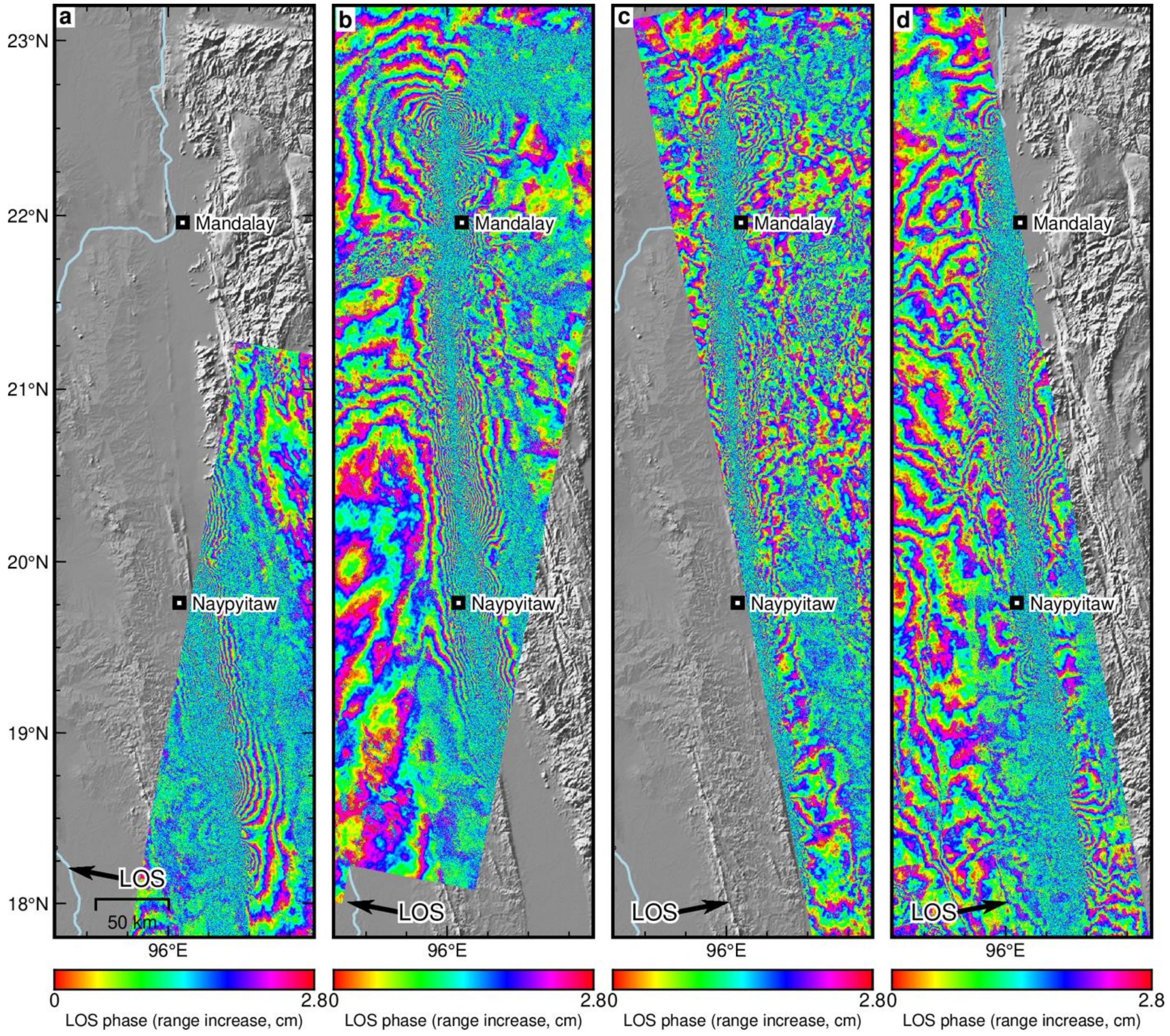

Supplementary Figure S2. Wrapped phase for all coseismic interferograms: (a) Descending track 33, 2025/03/19 - 2025/03/31; (b) Descending track 106, 2025/03/24 - 2025/04/05; (c) Ascending track 70, 2025/03/22 - 2025/04/03; (d) Ascending track 143, 2025/03/27 - 2025/04/08. Color change from red to yellow to blue indicates range increase (motion away from the satellite) along the radar line-of-sight (black arrows). One complete color cycle represents an additional 2.8 cm of range increase. Figure created using Generic Mapping Tools software[1] with hillshade derived from Shuttle Radar Topography Mission data[2,3]. Contains modified Copernicus Sentinel data [2025].

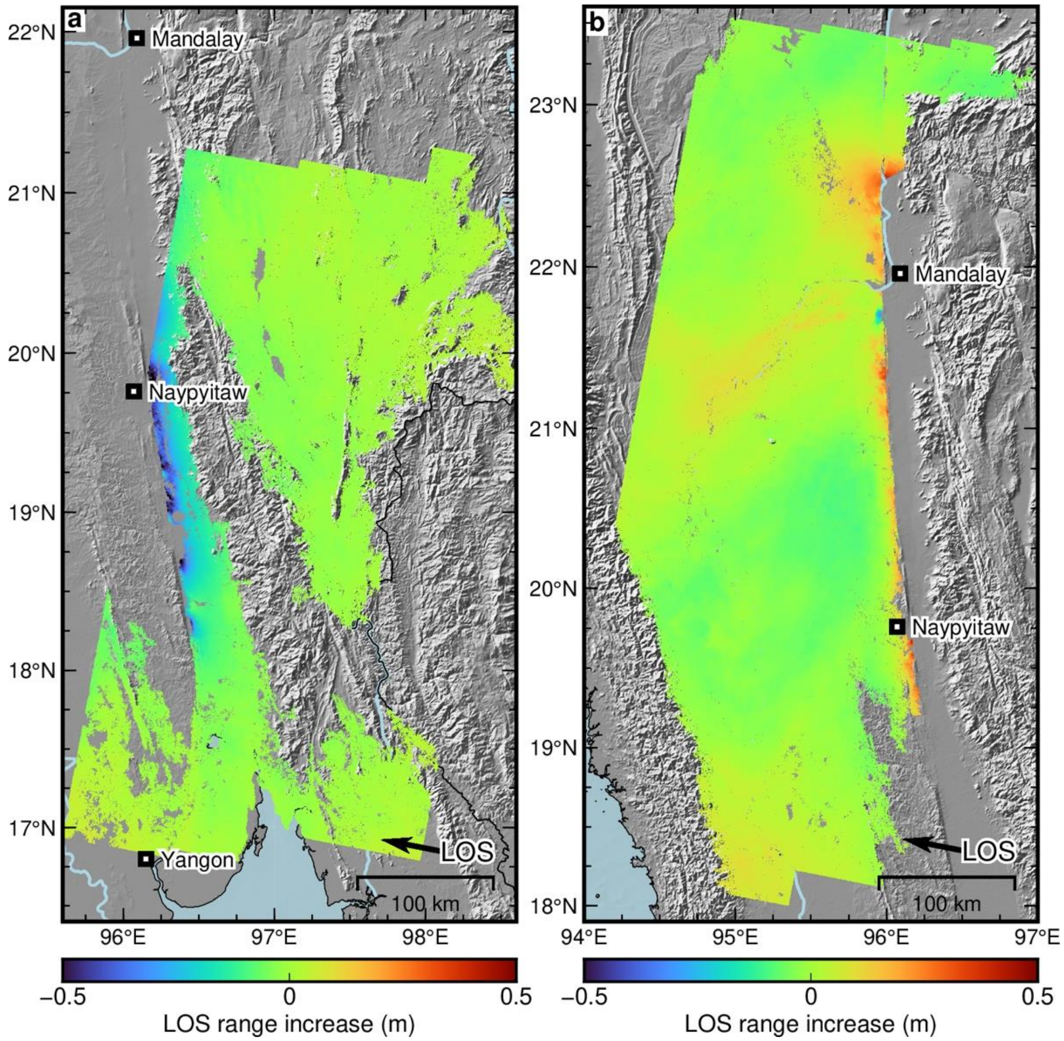

Supplementary Figure S3. Unwrapped, masked phase for descending coseismic interferograms used in the modeling: (a) track 33, 2025/03/19 - 2025/03/31; (b) track 106, 2025/03/24 - 2025/04/05. Positive values indicate motion away from the satellite along the radar line-of-sight direction (black arrows). Figure created using Generic Mapping Tools software[1] with hillshade derived from Shuttle Radar Topography Mission data[2,3]. Contains modified Copernicus Sentinel data [2025].

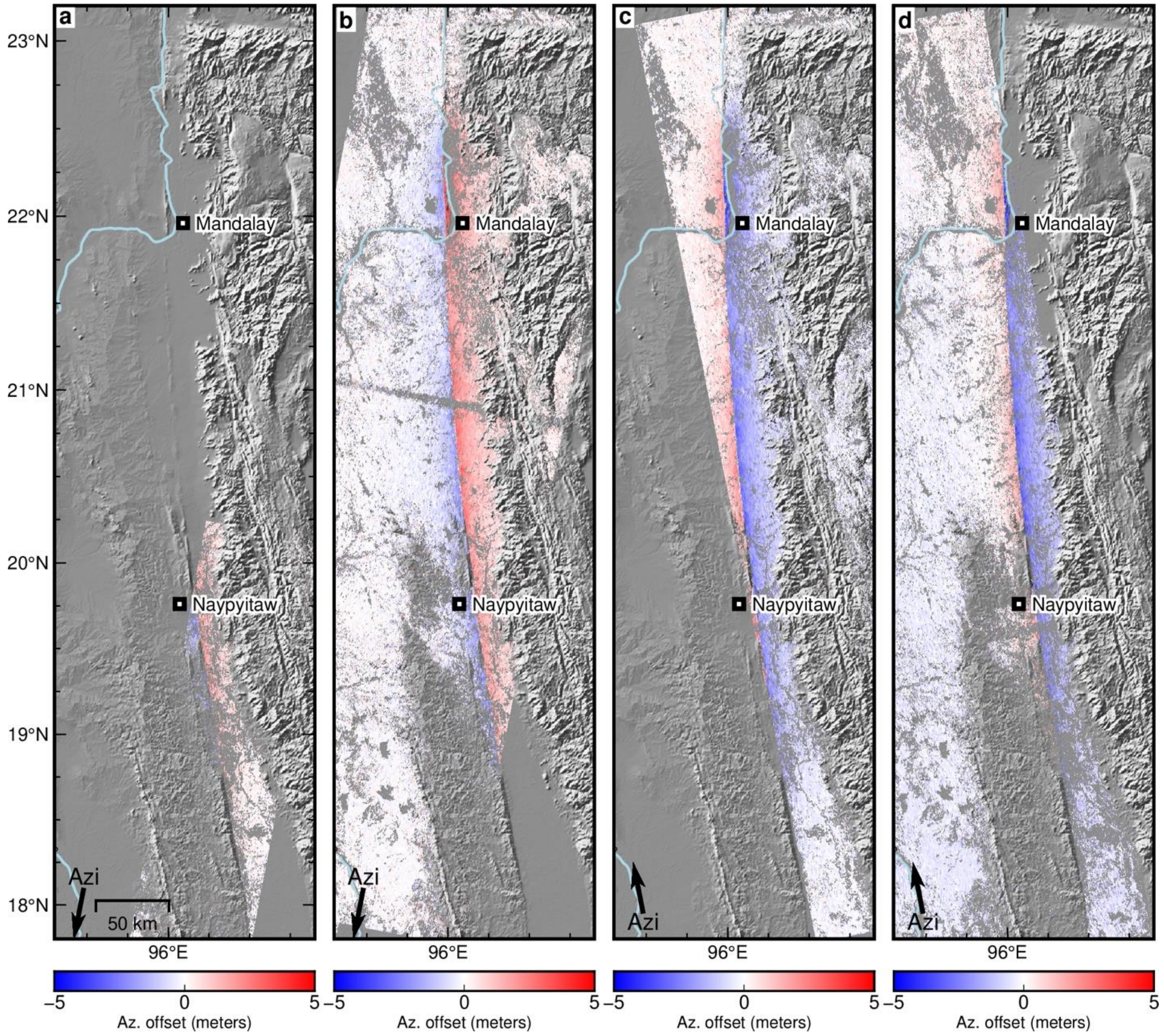

Supplementary Figure S4. Azimuth offsets for all coseismic pairs: (a) Descending track 33, 2025/03/19 - 2025/03/31; (b) Descending track 106, 2025/03/24 - 2025/04/05; (c) Ascending track 70, 2025/03/22 - 2025/04/03; (d) Ascending track 143, 2025/03/27 - 2025/04/08. Positive values indicate displacement along the satellite flight direction (black arrows). Figure created using Generic Mapping Tools software[1] with hillshade derived from Shuttle Radar Topography Mission data[2,3]. Contains modified Copernicus Sentinel data [2025].

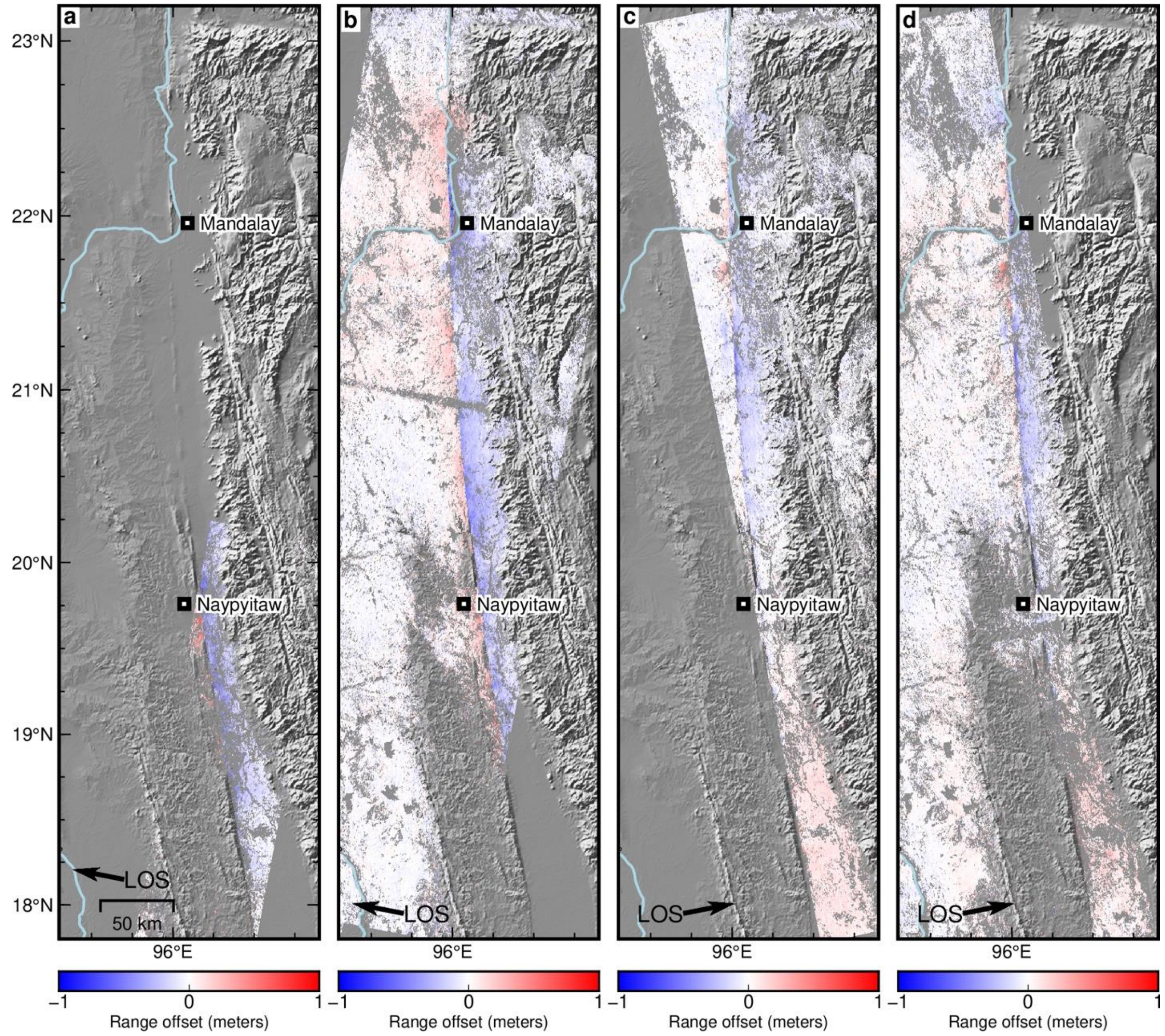

Supplementary Figure S5. Range offsets for all coseismic pairs: (a) Descending track 33, 2025/03/19 - 2025/03/31; (b) Descending track 106, 2025/03/24 - 2025/04/05; (c) Ascending track 70, 2025/03/22 - 2025/04/03; (d) Ascending track 143, 2025/03/27 - 2025/04/08. Positive values indicate motion away from the satellite along the radar line-of-sight direction (black arrows). Figure created using Generic Mapping Tools software with hillshade derived from Shuttle Radar Topography Mission data[2,3]. Contains modified Copernicus Sentinel data [2025].

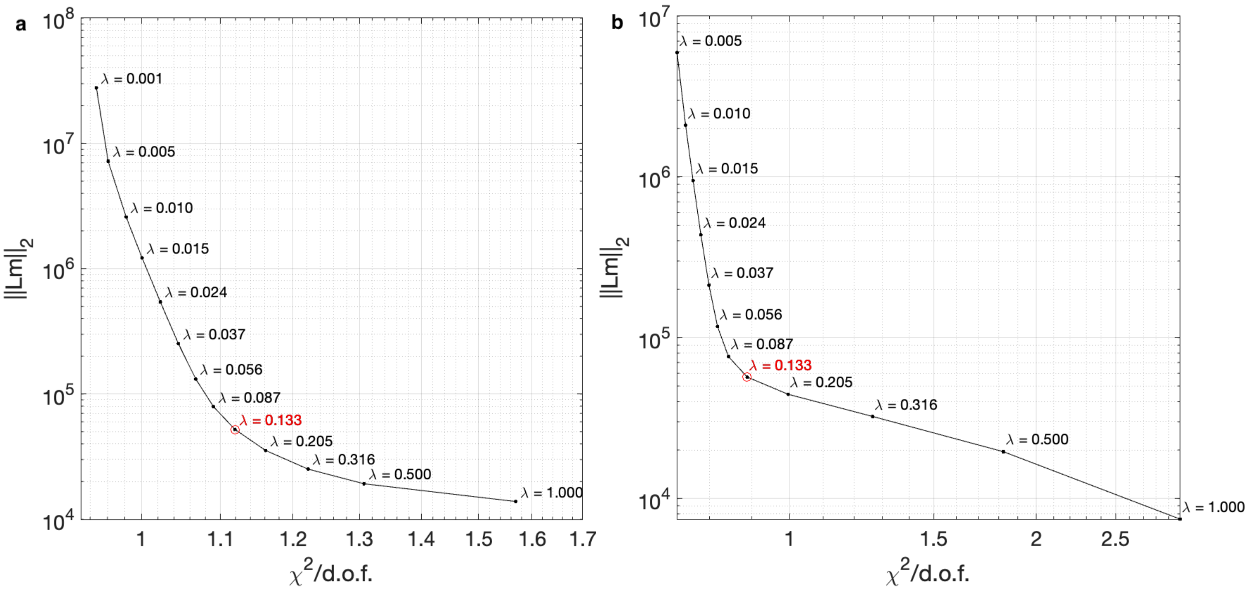

Supplementary Figure S6. L-curves for choice of model regularization (smoothing) parameter lambda. We compare the chi-squared per degree of freedom to the L-2 norm of the model roughness (L*m) on a log scale, and choose the point close to the highest curvature. Panel (a) shows the result for a vertical fault with no model norm penalty, (b) shows the result for a dipping fault and including a model norm penalty (see methods). The final selected value of lambda in both cases is 0.133, indicated in red in both panels.

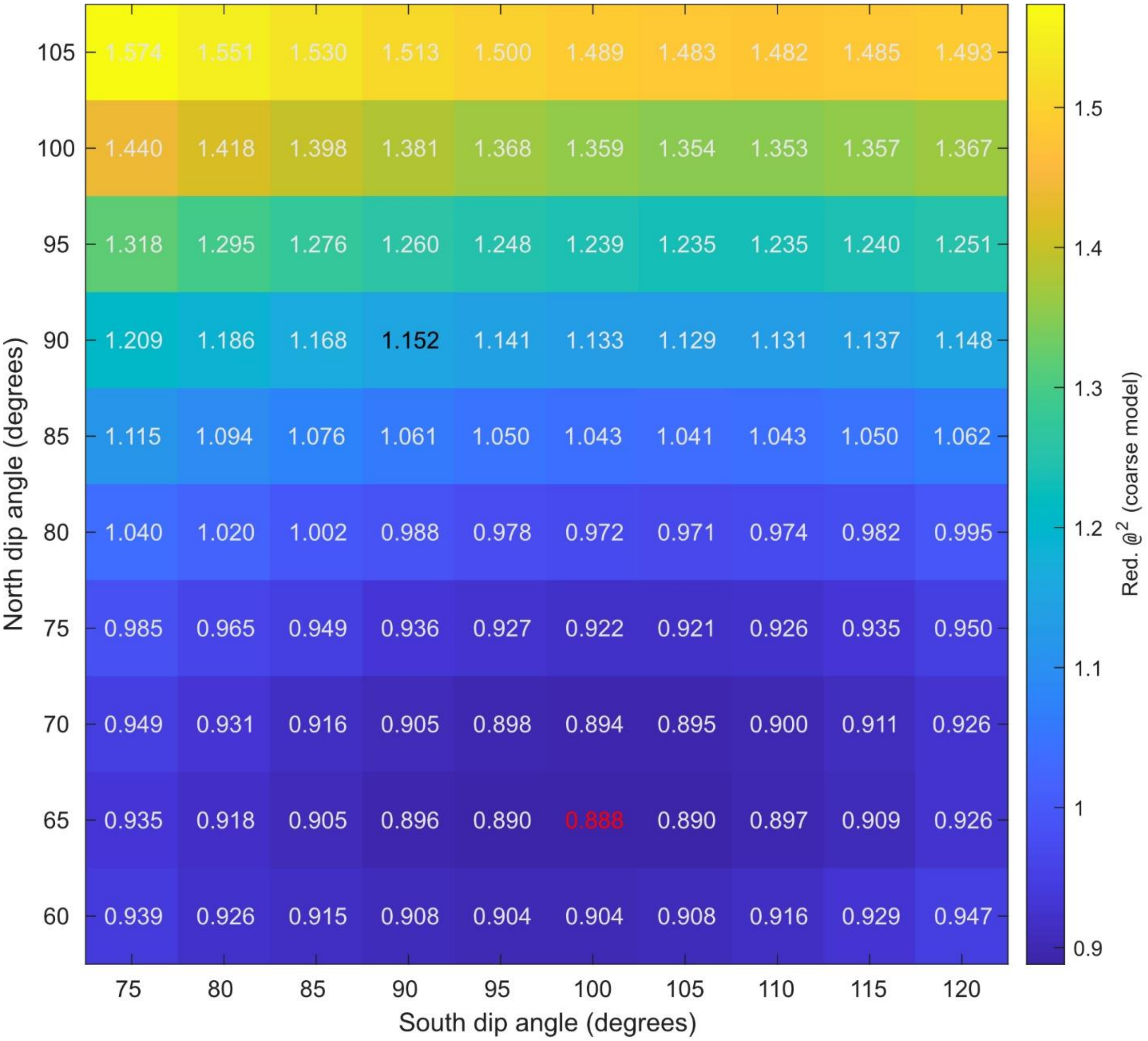

Supplementary Figure S7. Grid search for south dip angle (horizontal axis) and north dip angle (vertical axis), with a linear variation in dip angle assumed across a 200km length centered at 20ºN. The best-fitting model has a dip of 65º eastward in the northern section and 100º eastward (i.e., 80º westward) in the southern section (reduced chi-square value 0.888, labeled in red) and is shown in map view in Figure S8. The vertical model's reduced chi-square value is significantly higher (1.152) and is labeled in black.

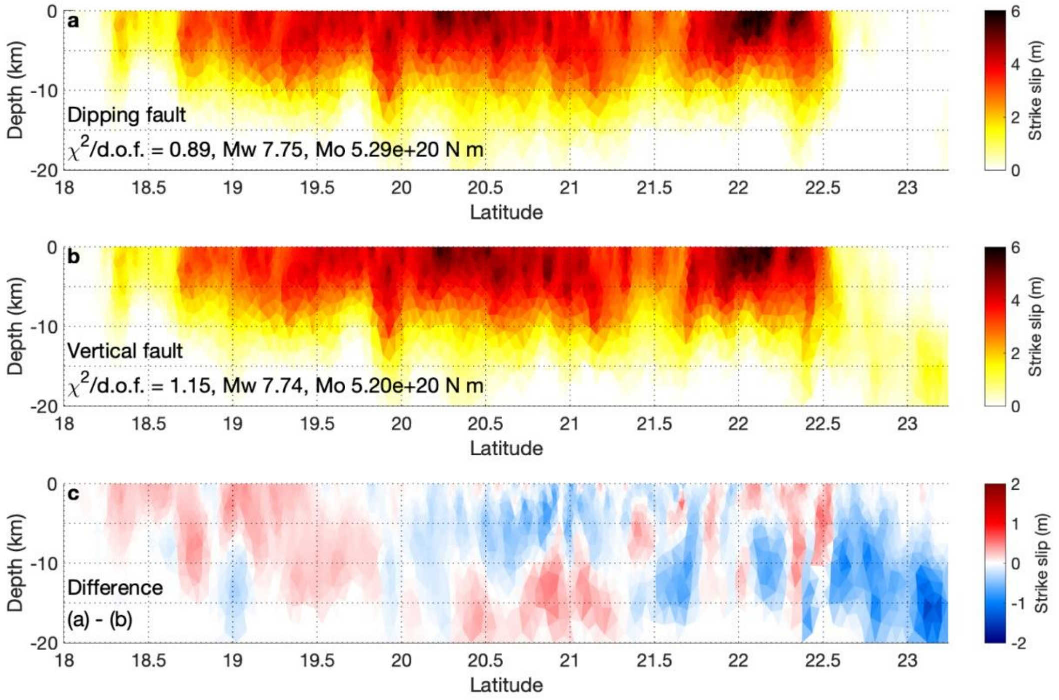

Supplementary Figure S8. Comparison of our preferred dipping model (b) with a vertical model (a). The difference is shown in (c). The two models are visually similar and have a nearly identical moment release (the dipping fault model is greater by 0.09 x 10^20 N m). Compared to the vertical model, the dipping model is more compact and has slightly less slip in the northern part, but more slip in the southern part.

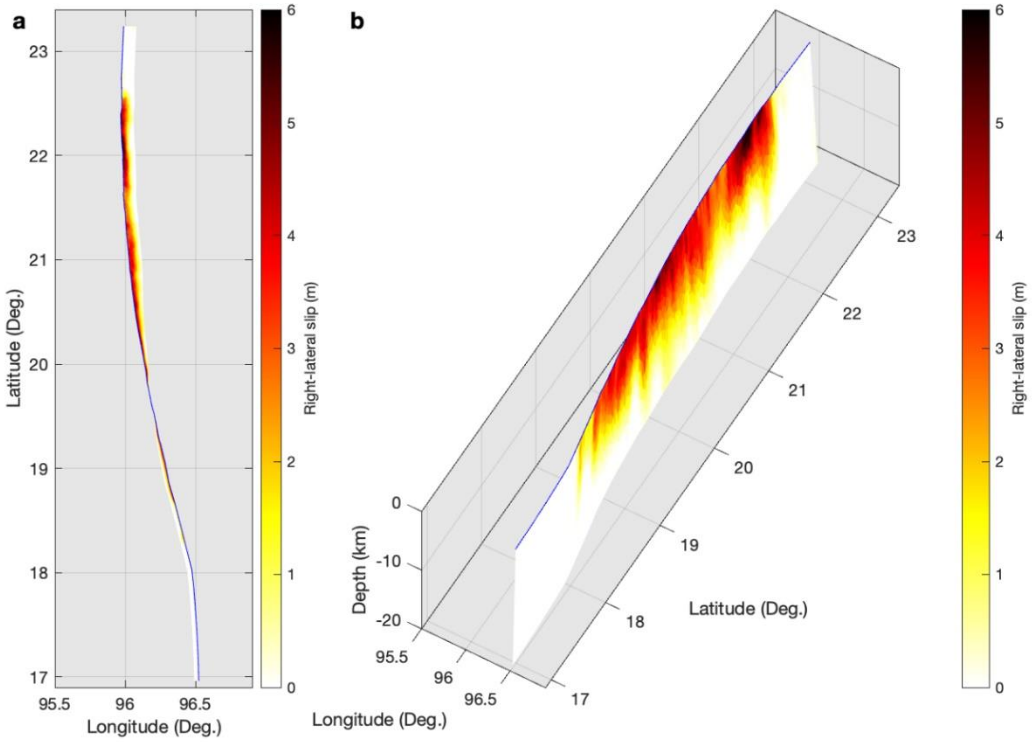

Supplementary Figure S9. Map view (a) and perspective view (b) showing the best-fitting dipping model, with the surface trace indicated in blue. The fault dips east at 65º north of 21ºN and west at 80º south of 19ºN, with a linear transition assumed in between.

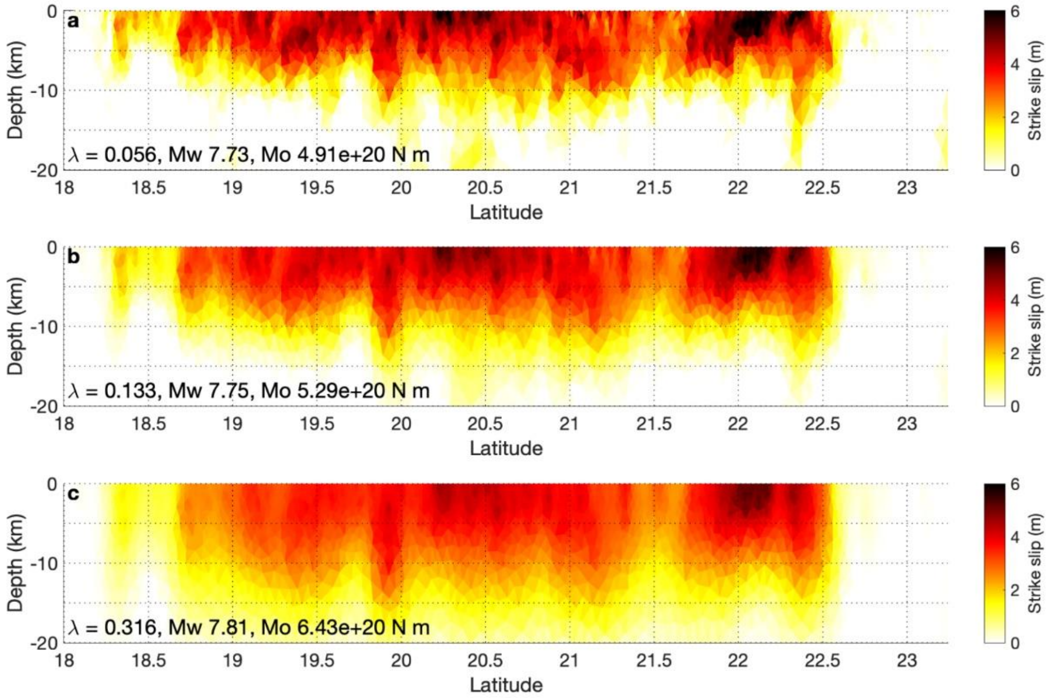

Supplementary Figure S10. Comparison of a model with less smoothing (a), our preferred model (b), and one with more smoothing (c). Model regularization parameter lambda and the total seismic moment (units of N m) and magnitude (assuming shear modulus of 30 GPa) are indicated in each panel. The overall pattern of displacements is similar in all three cases, but models with less smoothing have a smaller average depth of slip, suggesting that the true rupture depth might be relatively shallow.

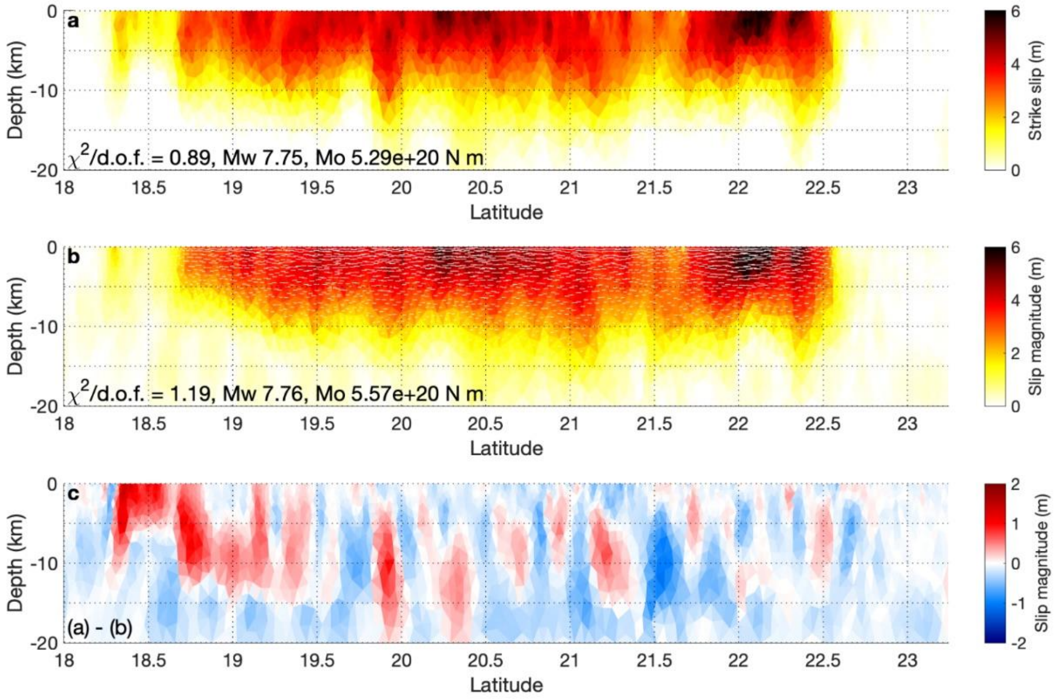

Supplementary Figure S11. Comparison of our preferred model (a) against a model with the same fault geometry but two components of slip (b); the difference is plotted in (c). The slip orientation on each patch is indicated as white lines in (b); the slip- and area-weighted rake of the overall two-component model is 182.6º, very close to pure right-lateral strike slip. The reduced chi-squared value increases significantly when two components of slip are permitted due to the larger number of degrees of freedom, indicating that the more complex model did not significantly improve the model's fit to the data. Referring to the models as 1D (strike slip only) and 2D (both slip components), we compute a two-parameter F-test defined as $F = (\,(\chi^2_{1D} - \chi^2_{2D})/(dof_{1D} - dof_{2D})\,)\;/\;(\chi^2_{2D}/dof_{2D})$. The result is F = 0.35 compared to an expected range of 0.95 – 1.05, which allows us to reject the more complex model with very high confidence (1-*p* = 10^-275).

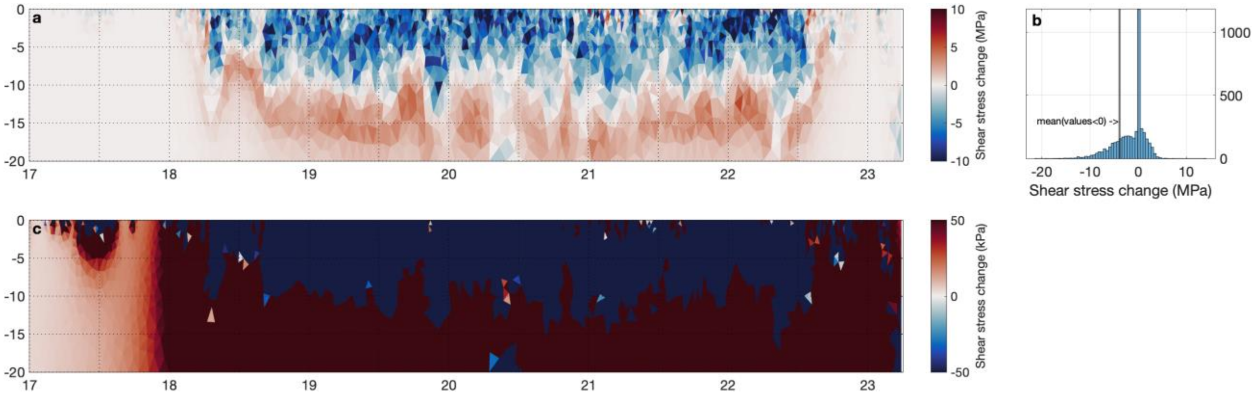

Supplementary Figure S12. Shear stress change on the fault for our preferred model, in units of MPa (a). Histogram of all stress changes (b) indicates a mean value for patches with negative shear stress (slipping patches) of -4.1 MPa. (c) shows the same values as (a) in kPa, with an expanded color scale. The previous epicenter of the May 1930 earthquake (around 17.8ºN) has an increased shear stress between 10-50 kPa.

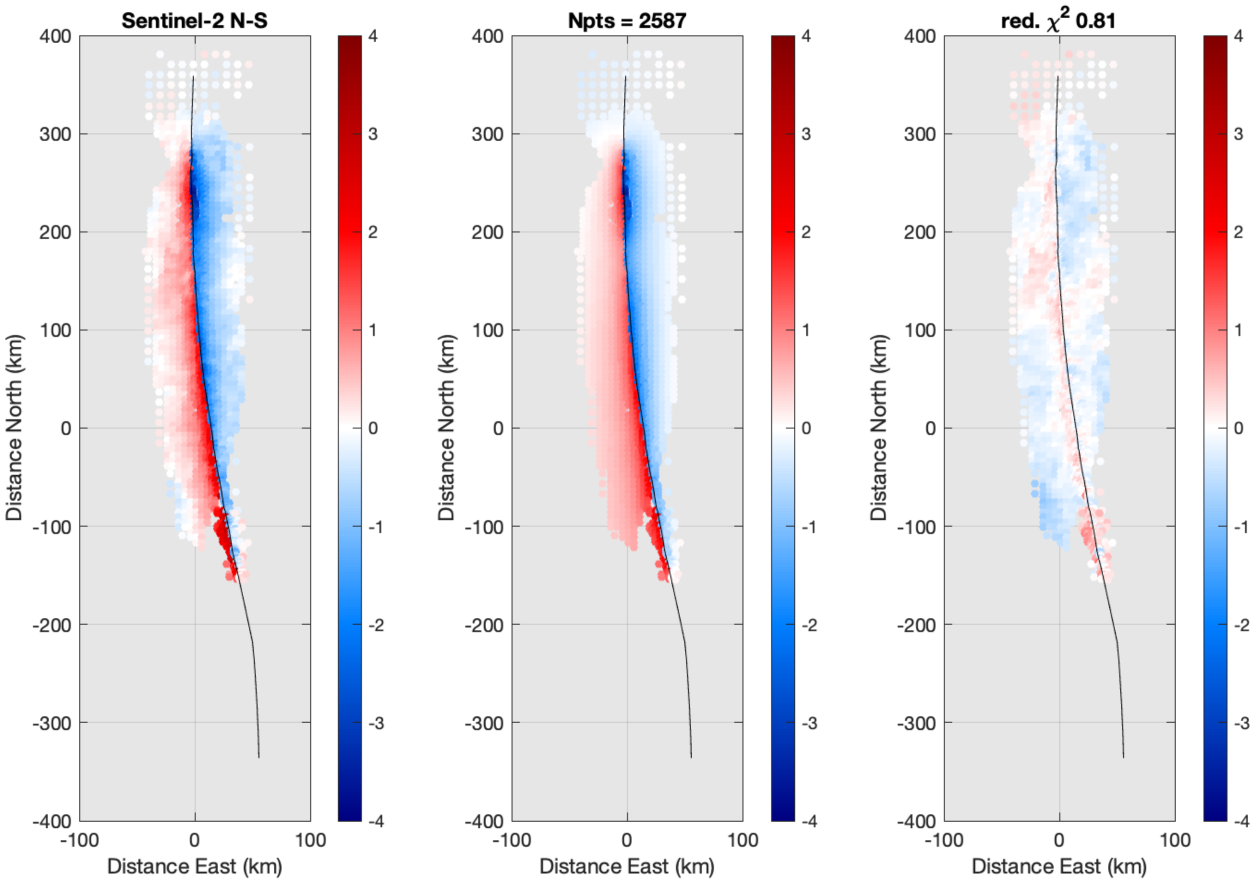

Supplementary Figure S13. Downsampled dataset, model predictions and residuals for Sentinel-2 North-South pixel offsets. (a) Downsampled dataset; (b) Model prediction; (c) Residuals (data - model). Number of data points is shown at the top of panel (b), and reduced chi-squared at the top of panel (c), calculated as as $(\mathbf{d}-\mathbf{Gm})^{\mathrm{T}}\,\mathbf{C}^{-1}\,(\mathbf{d}-\mathbf{Gm})/(N-P_{eff})$. The effective number of parameters relevant to this dataset (P_{eff}) is calculated as the sum of the N elements along the diagonal of the data influence (or projection) matrix $\widehat{\mathbf{H}}=\mathbf{G}(\mathbf{G}^{\mathrm{T}}\mathbf{G})^{-1}\mathbf{G}$, in the rows relevant to this dataset - i.e. in this case, the first 2587 elements. Map distances are shown in km relative to (96ºE, 20ºN), computed using the polyconic projection.

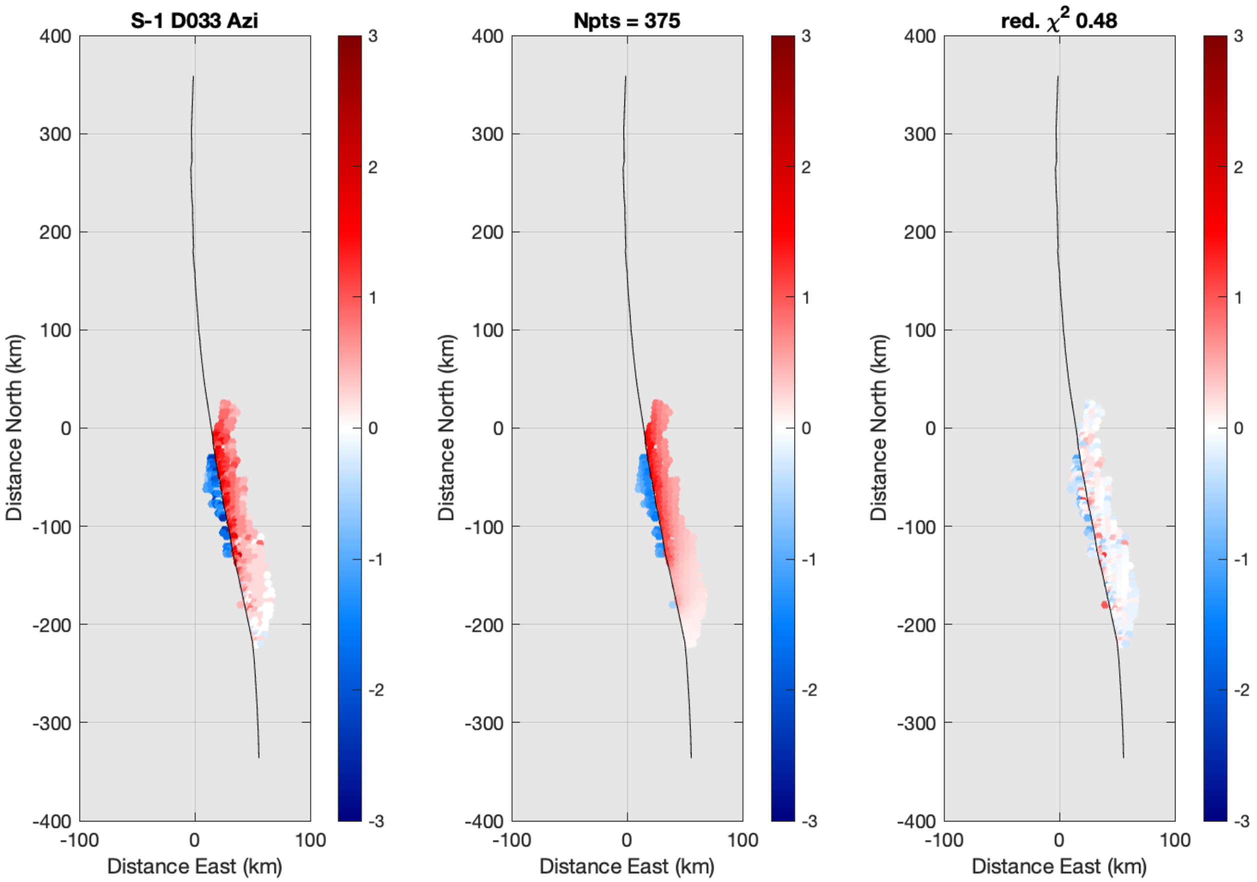

Supplementary Figure S14. Same as Supplementary Figure S10, for Sentinel-1 Azimuth offsets from descending track 33, 2025/03/19 - 2025/03/31.

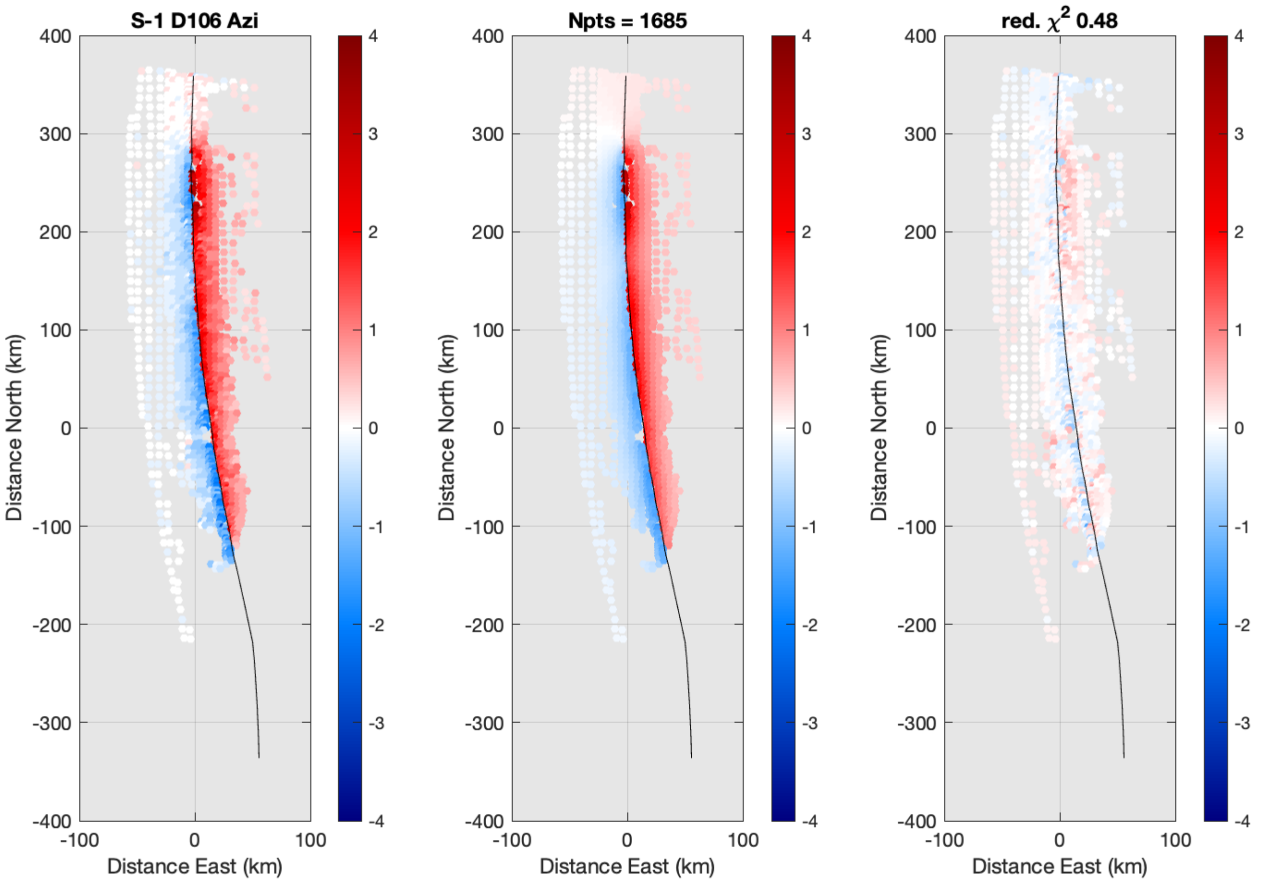

Supplementary Figure S15. Same as Supplementary Figure S10, for Sentinel-1 Azimuth offsets from descending track 106, 2025/03/24 - 2025/04/05.

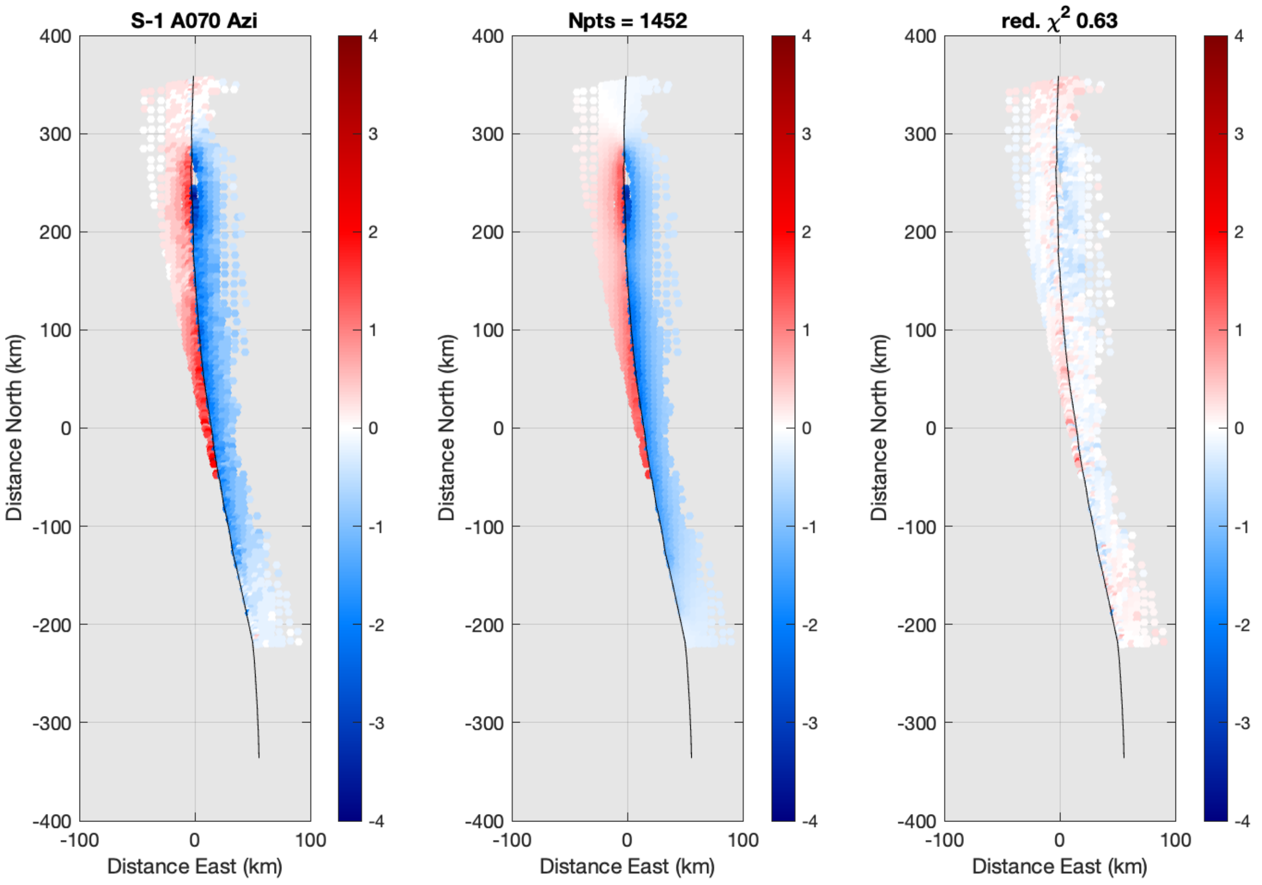

Supplementary Figure S16. Same as Supplementary Figure S10, for Sentinel-1 Azimuth offsets from ascending track 70, 2025/03/22 - 2025/04/03.

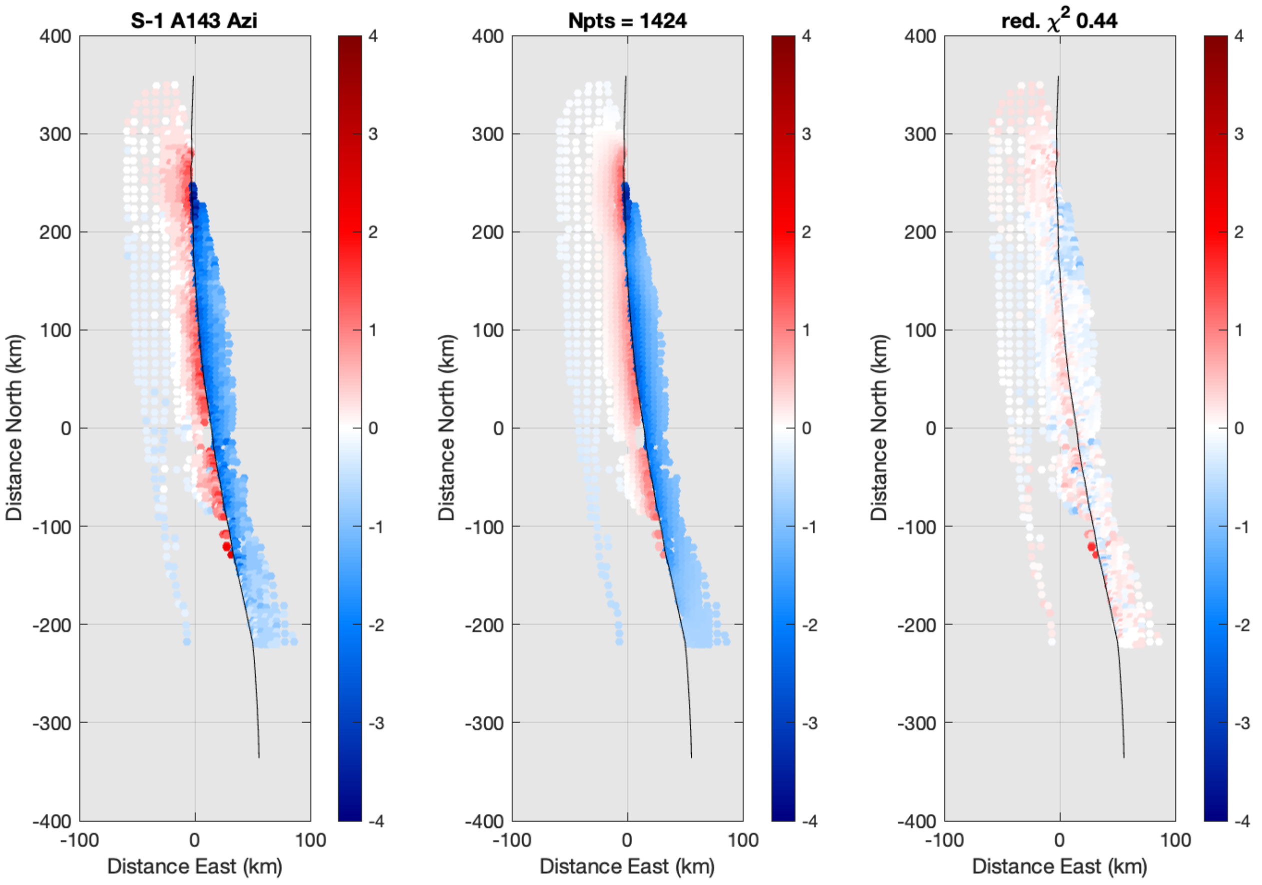

Supplementary Figure S17. Same as Supplementary Figure S10, for Sentinel-1 Azimuth offsets from ascending track 143, 2025/03/27 - 2025/04/08.

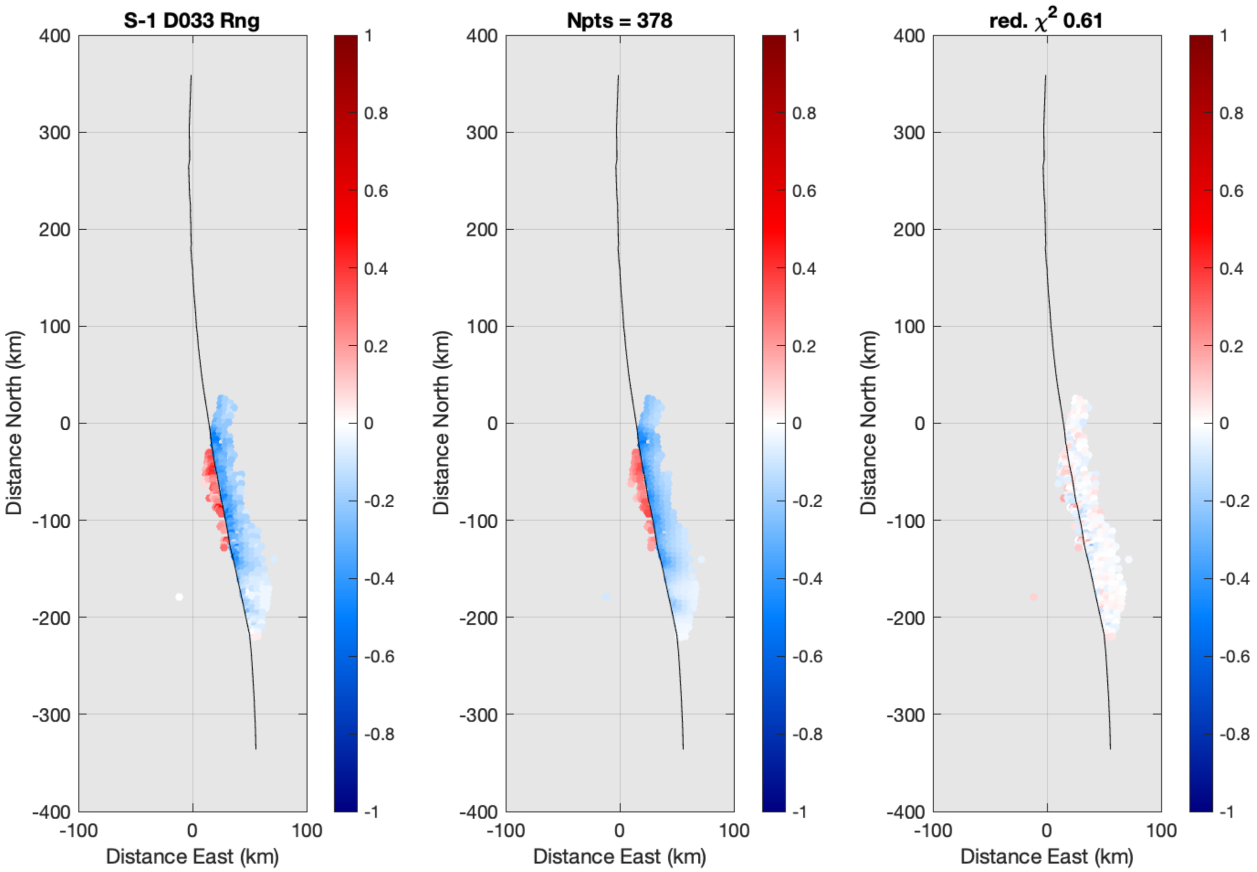

Supplementary Figure S18. Same as Supplementary Figure S10, for Sentinel-1 Range offsets from descending track 33, 2025/03/19 - 2025/03/31.

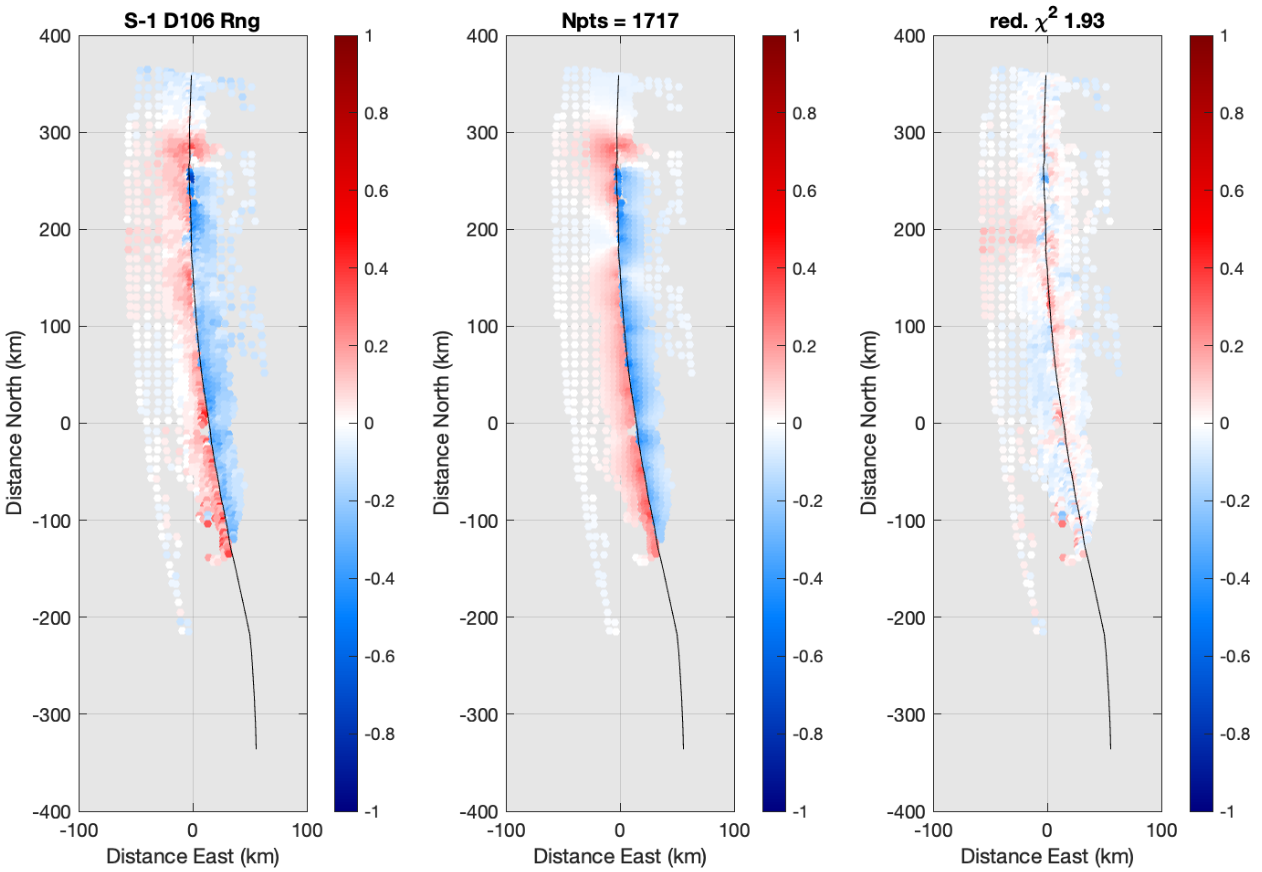

Supplementary Figure S19. Same as Supplementary Figure S10, for Sentinel-1 Range offsets from descending track 106, 2025/03/24 - 2025/04/05.

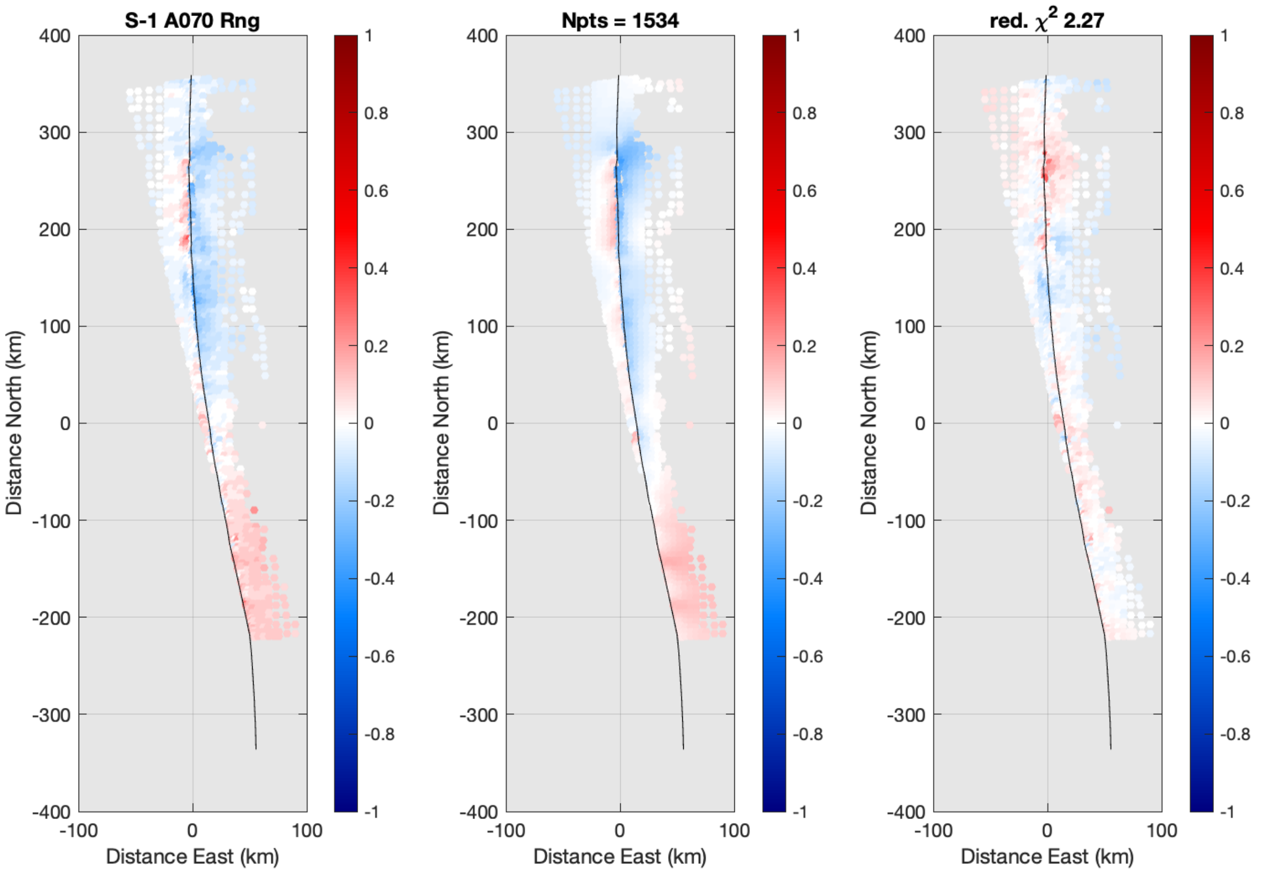

Supplementary Figure S20. Same as Supplementary Figure S10, for Sentinel-1 Range offsets from ascending track 70, 2025/03/22 - 2025/04/03.

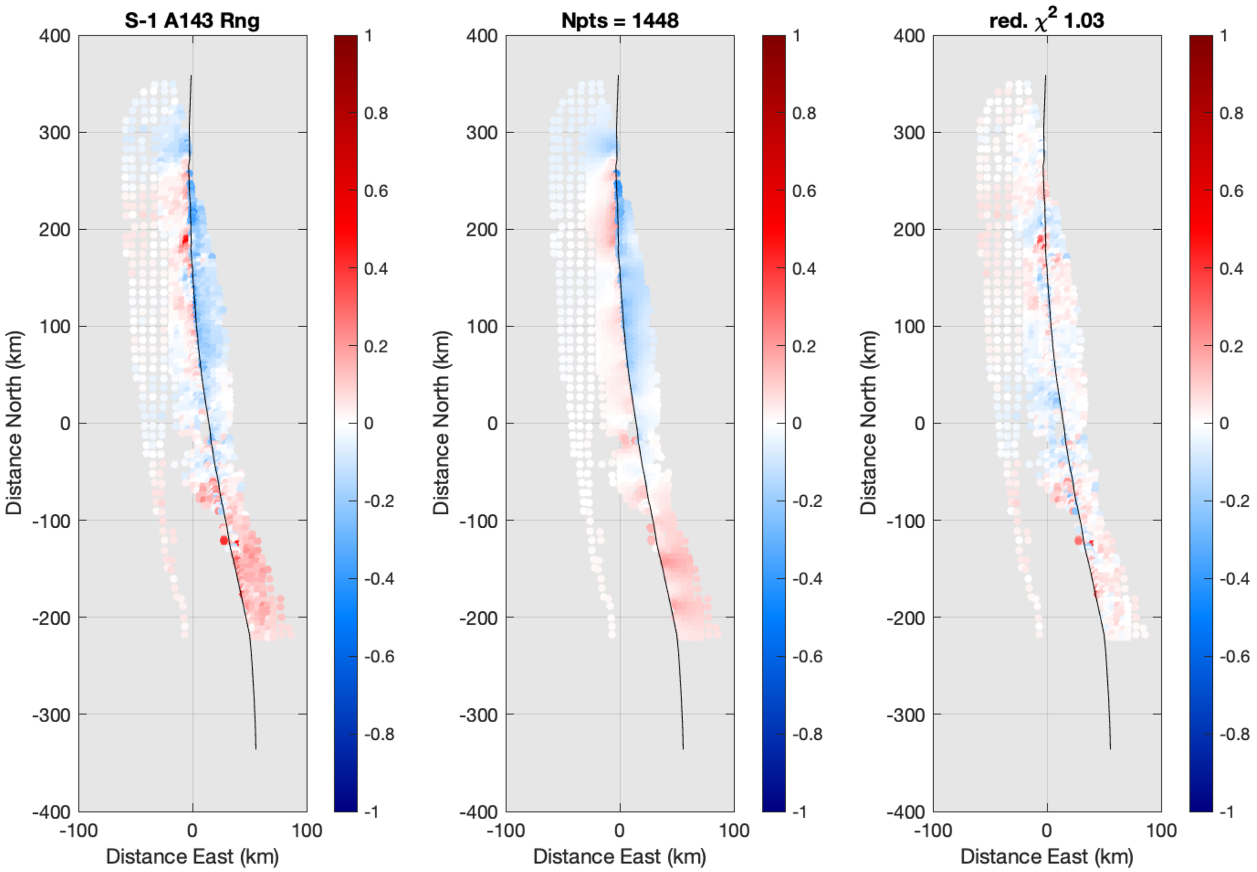

Supplementary Figure S21. Same as Supplementary Figure S10, for Sentinel-1 Range offsets from ascending track 143, 2025/03/27 - 2025/04/08.

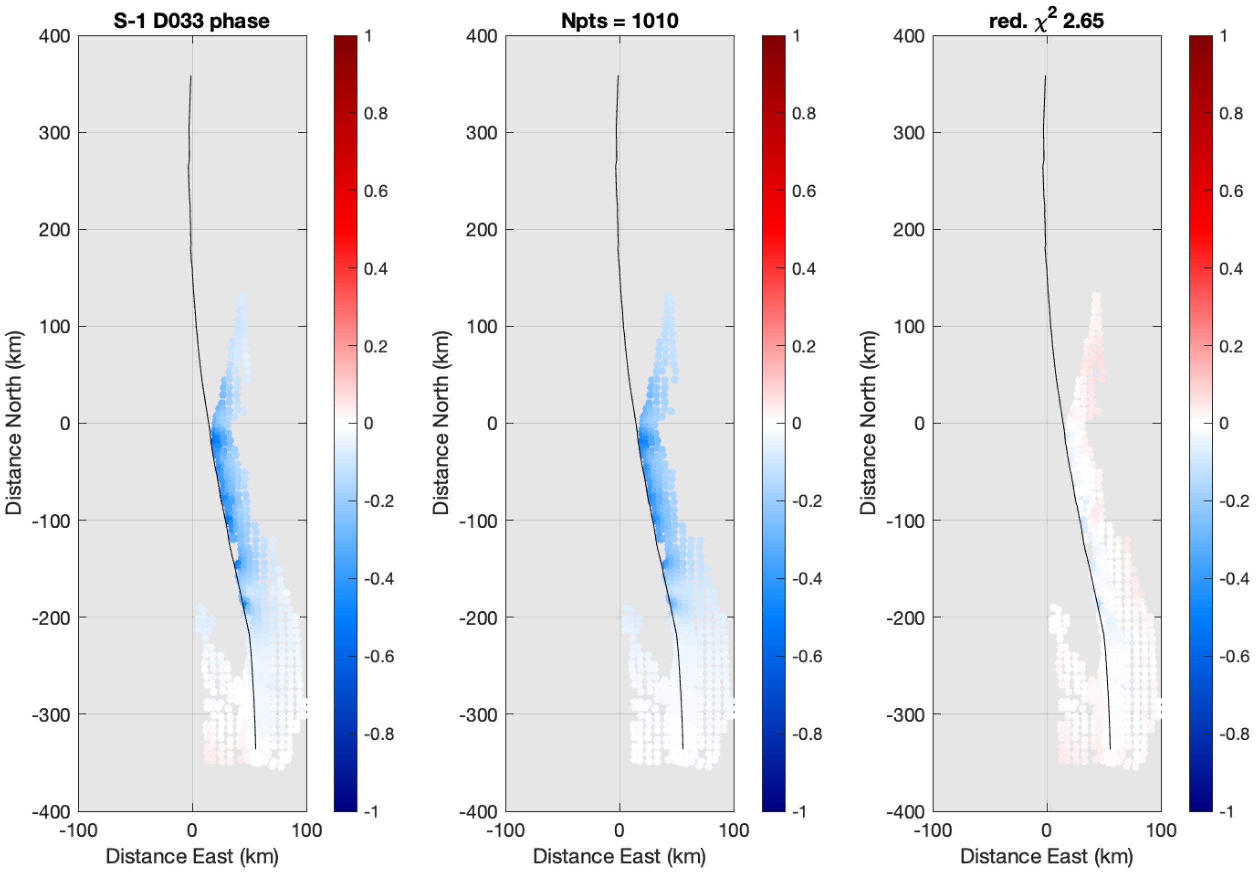

Supplementary Figure S22. Same as Supplementary Figure S10, for Sentinel-1 unwrapped phase from descending track 33, 2025/03/19 - 2025/03/31.

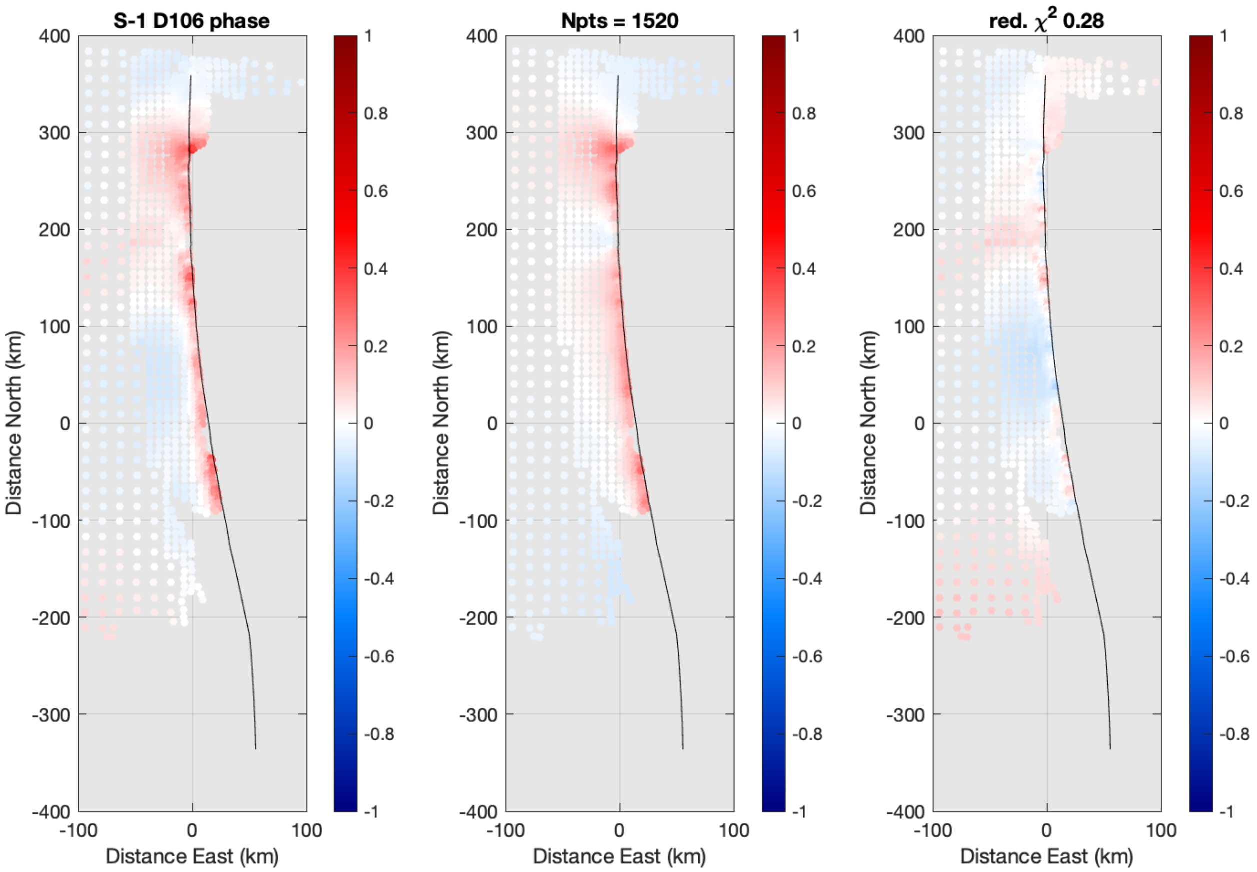

Supplementary Figure S23. Same as Supplementary Figure S10, for Sentinel-1 unwrapped phase from descending track 106, 2025/03/24 - 2025/04/05.

Supplementary Table S1. Values and references[4–29] for coseismic shallow slip deficit (SSD) and cumulative fault offset, used in Figure 5.

Earthquake	Mag.	Cumulative Fault Slip (km)	Shallow Slip Deficit (%)	Cumulative Offset Reference	Shallow Slip Deficit Reference
1992 Landers	7.3	3.5 - 4.6	18 - 46	Jachens et al., 2002	Xu et al., 2016
1999 Hector Mine	7.1	2.6 - 3.4	3 - 18	Jachens et al., 2002, Witkosky et al., 2020	Xu et al., 2016
1999 Izmit	7.6	52 - 70	0 - 4	Armijo et al., 1999, Akbayram et al., 2016	Çakir et al., 2003
2001 Kokoxili	7.8	80-120	~0	Fu and Awata, 2007	Antoine et al., 2024
2002 Denali	7.9	134 - 480	0 - 8	Miller et al., 2002, Waldien et al., 2021	Elliott et al., 2007, Wright et al., 2004
2013 Balochistan	7.7	5.6 - 11	~0 - 12	Zinke et al., 2014	Avouac et al., 2014, Lauer et al., 2020
2015 Sarez	7.2	-	22	-	Jin et al., 2022
2016 Kumamoto	7	0.8 - 1.4	2 - 15	Xi et al., 2025	Milliner et al., 2020
2019 Ridgecrest	7.1	0.5 - 1.6	27 - 33	Antoine et al., 2024	Jin and Fialko, 2020
2021 Maduo	7.4	4 - 5	17 - 30	Jin and Fialko, 2021, Zhao et al., 2023	Li et al., 2023, Sethanant et al., 2023
2023 Kahramanmaraş	7.8	19 - 25	6 - 25	Duman and Emre, 2013	Barbot et al., 2023, Wang et al., 2024
2010 El Mayor Cucapah	7.2	~1 - 4.4	11 - 60	Dorsey et al. 2012, Fletcher et al., 2020	Xu et al., 2016
2025 Sagaing	7.7	203 - 460	~0	Hla Maung, 1987, Myint Thein et al., 1991	**This study**

References

1. Farr, T. G. *et al.* The Shuttle Radar Topography Mission. *Rev. Geophys.* **45**, (2007).
2. Farr, T. G. & Kobrick, M. Shuttle radar topography mission produces a wealth of data. *Eos Trans. Am. Geophys. Union* **81**, 583–585 (2000).
3. Akbayram, K., Sorlien, C. C. & Okay, A. I. Evidence for a minimum 52±1km of total offset along the northern branch of the North Anatolian Fault in northwest Turkey. *Tectonophysics* **668–669**, 35–41 (2016).
4. Antoine, S. L., Klinger, Y., Wang, K. & Bürgmann, R. Coseismic Shallow Slip Deficit Accounted for by Diffuse Off-Fault Deformation. *Geophys. Res. Lett.* **51**, e2024GL110798 (2024).
5. Armijo, R., Meyer, B., Hubert, A. & Barka, A. Westward propagation of the North Anatolian fault into the northern Aegean: Timing and kinematics. *Geology* **27**, 267–270 (1999).
6. Avouac, J.-P. *et al.* The 2013, Mw 7.7 Balochistan earthquake, energetic strike-slip reactivation of a thrust fault. *Earth Planet. Sci. Lett.* **391**, 128–134 (2014).
7. Barbot, S. *et al.* Slip distribution of the February 6, 2023 Mw 7.8 and Mw 7.6, Kahramanmaraş, Turkey earthquake sequence in the East Anatolian Fault Zone. *Seismica* **2**, (2023).
8. Çakir, Z. *et al.* Coseismic and early post-seismic slip associated with the 1999 Izmit earthquake (Turkey), from SAR interferometry and tectonic field observations. *Geophys. J. Int.* **155**, 93–110 (2003).
9. Dorsey, R. J., Axen, G. J., Peryam, T. C. & Kairouz, M. E. Initiation of the Southern Elsinore Fault at ~1.2 Ma: Evidence from the Fish Creek–Vallecito Basin, southern California. *Tectonics* **31**, (2012).

10. Duman, T. Y. & Emre, Ö. The East Anatolian Fault: geometry, segmentation and jog characteristics. in *Geological Development of Anatolia and the Easternmost Mediterranean Region* (eds Robertson, A. H. F., Parlak, O. & Ünlügenç, U. C.) vol. 372 0 (Geological Society of London, 2013).

11. Elliott, J. L., Freymueller, J. T. & Rabus, B. Coseismic deformation of the 2002 Denali fault earthquake: Contributions from synthetic aperture radar range offsets. *J. Geophys. Res. Solid Earth* **112**, (2007).

12. Fletcher, J. M. *et al.* An analysis of the factors that control fault zone architecture and the importance of fault orientation relative to regional stress. *GSA Bull.* **132**, 2084–2104 (2020).

13. Fu, B. & Awata, Y. Displacement and timing of left-lateral faulting in the Kunlun Fault Zone, northern Tibet, inferred from geologic and geomorphic features. *J. Asian Earth Sci.* **29**, 253–265 (2007).

14. Jachens, R. C., Langenheim, V. E. & Matti, J. C. Relationship of the 1999 Hector Mine and 1992 Landers Fault Ruptures to Offsets on Neogene Faults and Distribution of Late Cenozoic Basins in the Eastern California Shear Zone. *Bull. Seismol. Soc. Am.* **92**, 1592–1605 (2002).

15. Jin, Z. & Fialko, Y. Finite Slip Models of the 2019 Ridgecrest Earthquake Sequence Constrained by Space Geodetic Data and Aftershock Locations. *Bull. Seismol. Soc. Am.* **110**, 1660–1679 (2020).

16. Jin, Z. & Fialko, Y. Coseismic and Early Postseismic Deformation Due to the 2021 M7.4 Maduo (China) Earthquake. *Geophys. Res. Lett.* **48**, e2021GL095213 (2021).

17. Jin, Z., Fialko, Y., Zubovich, A. & Schöne, T. Lithospheric Deformation Due To the 2015 M7.2 Sarez (Pamir) Earthquake Constrained by 5 years of Space Geodetic Observations. *J. Geophys. Res. Solid Earth* **127**, e2021JB022461 (2022).

18. Lauer, B., Grandin, R. & Klinger, Y. Fault Geometry and Slip Distribution of the 2013 Mw 7.7 Balochistan Earthquake From Inversions of SAR and Optical Data. *J. Geophys. Res. Solid Earth* **125**, e2019JB018380 (2020).

19. Maung, H. Transcurrent movements in the Burma–Andaman Sea region. *Geology* **15**, 911–912 (1987).

20. Miller, M. L., Bradley, D. C., Bundtzen, T. K. & McClelland, W. Late Cretaceous through Cenozoic Strike-Slip Tectonics of Southwestern Alaska. *J. Geol.* **110**, 247–270 (2002).

21. Milliner, C., Bürgmann, R., Inbal, A., Wang, T. & Liang, C. Resolving the Kinematics and Moment Release of Early Afterslip Within the First Hours Following the 2016 Mw 7.1 Kumamoto Earthquake: Implications for the Shallow Slip Deficit and Frictional Behavior of Aseismic Creep. *J. Geophys. Res. Solid Earth* **125**, e2019JB018928 (2020).

22. Myint Thein. On the lateral displacement of Sagaing fault. *Georeports Yangon Univ.* **1**, 23–34 (1991).

23. Waldien, T. S., Roeske, S. M. & Benowitz, J. A. Tectonic Underplating and Dismemberment of the Maclaren-Kluane Schist Records Late Cretaceous Terrane Accretion Polarity and 480 km of Post-52 Ma Dextral Displacement on the Denali Fault. *Tectonics* **40**, e2020TC006677 (2021).

24. Wang, K., Xu, X. & Hu, Y. Kinematics of the 2023 Kahramanmaraş Earthquake Doublet: Biased Near-Fault Data and Shallow Slip Deficit. *Seismol. Res. Lett.* **96**, 828–837 (2024).

25. Witkosky, R. A. *et al.* The Lavic Lake Fault: A Long-Term Cumulative Slip Analysis via Combined Field Work and Thermal Infrared Hyperspectral Airborne Remote Sensing. *Remote Sens.* **12**, 3586 (2020).

26. Wright, T. J., Lu, Z. & Wicks, C. Constraining the Slip Distribution and Fault Geometry of the Mw 7.9, 3 November 2002, Denali Fault Earthquake with Interferometric Synthetic Aperture Radar and Global Positioning System Data. *Bull. Seismol. Soc. Am.* **94**, S175–S189 (2004).

27. Xu, X. *et al.* Refining the shallow slip deficit. *Geophys. J. Int.* **204**, 1867–1886 (2016).

28. Zhao, L. *et al.* Fault geometry and low frictional control of the near-field postseismic deformation of the 2021 Mw 7.3 Maduo earthquake. *Tectonophysics* **863**, 230000 (2023).
